# Identification and Expression Analysis of Diapause Hormone and Pheromone Biosynthesis Activating Neuropeptide (DH-PBAN) in the Legume Pod Borer, *Maruca vitrata* Fabricius

**DOI:** 10.1371/journal.pone.0084916

**Published:** 2014-01-07

**Authors:** Jian-Cheng Chang, Srinivasan Ramasamy

**Affiliations:** AVRDC – The World Vegetable Center, Tainan, Taiwan; University of Rouen, France

## Abstract

Neuropeptides play essential roles in a variety of physiological responses that contribute to the development and reproduction of insects. Both the diapause hormone (DH) and pheromone biosynthesis activating neuropeptide (PBAN) belong to the PBAN/pyrokinin neuropeptide family, which has a conserved pentapeptide motif FXPRL at the *C*-terminus. We identified the full-length cDNA encoding DH-PBAN in *Maruca vitrata*, a major lepidopteran pest of leguminous crops. The open reading frame of Marvi-DH-PBAN is 591 bp in length, encoding 197 amino acids, from which five putative neuropeptides [DH, PBAN, α-subesophageal ganglion neuropeptide (SGNP), β-SGNP and γ-SGNP] are derived. Marvi-DH-PBAN was highly similar (83%) to DH-PBAN of *Omphisa fuscidentalis* (Lepidoptera: Crambidae), but possesses a unique *C*-terminal FNPRL motif, where asparagine has replaced a serine residue present in other lepidopteran PBAN peptides. The genomic DNA sequence of Marvi-DH-PBAN is 6,231 bp in size and is composed of six exons. Phylogenetic analysis has revealed that the Marvi-DH-PBAN protein sequence is closest to its homolog in Crambidae, but distant from Diptera, Coleoptera and Hymenoptera DH-PBAN, which agrees with the current taxonomy. DH-PBAN transcripts were present in the head and thoracic complex, but absent in the abdomen of *M. vitrata*. Real-time quantitative PCR assays have demonstrated a relatively higher expression of Marvi-DH-PBAN mRNA in the latter half of the pupal stages and in adults. These findings represent a significant step forward in our understanding of the DH-PBAN gene architecture and phylogeny, and raise the possibility of using Marvi-DH-PBAN to manage *M. vitrata* populations through molecular techniques.

## Introduction

The life cycle of insects consists of different developmental stages as well as various physiological processes, such as metamorphosis, diapause, eclosion, mating and reproduction. Neuropeptides play vital regulatory roles in a wide range of these biological activities in insects [1]. Diapause hormone (DH) and pheromone biosynthesis activating neuropeptide (PBAN) are two critical neuropeptides encoded by a single precursor and belong to the PBAN/pyrokinin family, or the FXPRL peptide family [2], due to their conserved last five *C*-terminal sequence that is the minimum requirement for physiological activity [3]–[5]. In moths, the DH neuropeptide has been reported to trigger embryonic diapause or terminate pupal diapause, which is essential for survival under extreme or unfavourable circumstances, whereas PBAN functions in female sex pheromone biosynthesis. The first DH and PBAN proteins were identified and isolated from *Bombyx mori* [6] and *Helicoverpa zea* [7], respectively. Since then, DH-PBAN proteins have been reported for more than 30 insect species spanning four orders (see review by [8]).

The DH-PBAN precursor is cleaved to yield five neuropeptides, namely DH, PBAN, α-, β-, and γ-subesophageal ganglion neuropeptides (SGNP) in lepidopterans, while the α-SGNP is absent from Diptera, Hymenoptera, and Coleoptera DH-PBAN genes [9]. The five neuropeptides of lepidopterans are synthesized mainly in the subesophageal ganglia (SG), and are released into the hemolymph through the corpora cardiaca (see review by [10]). The full physiological functions of the DH-PBAN neuropeptides have yet to be elucidated, but PBAN/pyrokinin family peptides have been suggested to regulate the diapause and pheromone synthesis in moths, the myotropic function of hindgut in cockroaches [11], [12], the cuticular melanization in silkworm larvae [13], [14], and accelerate puparium formation in flies [15]. Furthermore, Choi and Vander Meer [16] reported that each PBAN/pyrokinin family peptide stimulates all listed physiological functions *in vitro*, and therefore additional functions are likely to be revealed from this broadly conserved peptide family in Insecta.

DH-PBAN has been widely studied in insects and great emphasis was given to taxa in the lepidopteran superfamilies Noctuoidea and Bombycoidea. There is no previous knowledge of the DH-PBAN genomic structure and gene expression profile in members of the Crambidae, an agriculturally and economically important family of Lepidoptera. The primary objective of the present study was to molecularly characterize DH-PBAN in a serious lepidopteran pest of leguminous crops, the legume pod borer *Maruca vitrata* Fabricius ( = *Maruca testulalis* Geyer). *M. vitrata* is a major agricultural and commercial pest in tropical and subtropical zones and reduces yield in various vegetable and grain legumes by up to 80% [17]–[20]. The increased and indiscriminate use of chemical insecticides to control *M. vitrata* reported in Asia, Africa, and Latin America has caused severe damage to the environment as well as to human health [21], [22]. Therefore it is imperative to develop alternative, environmentally friendly control methods such as pheromone traps to manage *M. vitrata*.

Variations in pheromone composition and preference could be a key to subvert the mating behavior of *M. vitrata* and achieve sustainable pest control. (E,E)-10,12-hexadecadienal, (E,E)-10,12-hexadecadienol, and (E)-10-hexadecenal are the major component and minor components of the *M. vitrata* sex pheromone, respectively [23], [24]. Re-examination of *M. vitrata* sex pheromone revealed the presence of two additional components, (*E*)-10-hexadecenol and (*Z,Z,Z,Z,Z*)-3,6,9,12,15-tricosapentaene [25]. Variations have been observed in the responses of *M. vitrata* males to synthetic sex pheromone lures over various geographical regions. A synthetic sex pheromone for *M. vitrata* consisting of (E,E)-10,12-hexadecadienal, (E,E)-10,12-hexadecadienol, and (E)-10-hexadecenal was developed and attracted male *M. vitrata* moths in Benin and Ghana, while (E,E)-10,12-hexadecadienal alone was most effective in Burkina Faso [26]. Neither pheromone was effective in Taiwan and Thailand, while a variant blend was effective in India [25]. These observed variations in the responses of male *M. vitrata* moths to the pheromone lures over different geographical regions indicated the possibility of the existence of polymorphism in the components of *M. vitrata* sex pheromones. Understanding the role of PBAN in relation to a specific pheromone compound in *M. vitrata* is important because sex pheromone biosynthesis in insects usually is regulated by PBAN. Hence, the first step is to expound the molecular level information of pheromone associated genes, especially the genotypic variation in PBAN among distinct *M. vitrata* populations. In the current study, we identified the structure of the DH-PBAN gene from *M. vitrata* (Marvi-DH-PBAN) and elucidated the genomic organization. The DH-PBAN-based phylogenetic tree was constructed among insect species. We also demonstrated the Marvi-DH-PBAN gene expression profiles at different developmental stages based on quantitative PCR analysis. This information opens the path to study PBAN diversity in *M. vitrata* populations to assess the role of this protein in the regulation of sex pheromone biosynthesis and composition.

## Materials and Methods

### Ethics Statement

No specific permits were required for the described field studies, and no specific permissions were required for these locations/activities. We confirm that samples were taken from non-endangered, non-protected species on open, public lands.

### Insects

*Maruca vitrata* larvae were collected from legume crops in Taiwan. Host legume leaves served as the insects’ diet until pupation. After pupation, each individual pupa was isolated, sexed and maintained in a plastic cup until eclosion. Adults were reared separately in wire-meshed acrylic cylinders (30 cm×15 cm) and provided with water-soaked sponges and a 10% honey solution. Insect samples were kept at 26°C with a photoperiod of 14∶10 (light:dark) commencing with the larval stage until their dissection. Heads, thoraces, and abdomens from one- to six-day-old pupae and one- to three-day-old adult moths of both sexes were dissected and preserved in RNAlater® solution (Ambion Inc., Austin, TX) for subsequent isolation of total RNA.

### RNA Extraction

Total RNA was isolated from homogenized heads, thoraces, and abdomens using Total RNA Mini Kit (Tissue) (Geneaid Biotech Ltd., Taipei, Taiwan) according to the manufacturer’s protocol, with in-column DNase I treatment (GMbiolab Co., Ltd., Taichung, Taiwan). RNA was quantified by absorbance measurement on a spectrophotometer at 260 nm, while the purity of the RNA was determined by the ratio of A_260_/A_280_.

### De novo Transcriptome Sequencing

The transcriptome sequencing was performed by Genomics BioSci & Tech Co. (Taipei, Taiwan), in order to identify the candidate DH-PBAN gene homologs in *M. vitrata*. In brief, mRNA was enriched from 15 µg total RNA isolated from 3-day-old adult female moths using magnetic oligo (dT) beads. mRNA was fragmented by divalent cations and heat treatment. Random hexamer-primers were used for first-strand cDNA synthesis and the second-strand cDNA was synthesized using RNase H, DNA polymerase I with buffer and dNTPs. Short fragments were purified with a QiaQuick PCR extraction kit (Qiagen, Valencia, CA, USA) and further subjected to end-repair and addition of a single (A) base. After that, the short fragments were ligated with sequencing adapters. By gel selection, suitable sizes of cDNA fragments were then PCR amplified as templates. Finally, the library was sequenced using Illumina HiSeq™ 2000 (BGI, Beijing, China).

All dirty raw reads from RNA-seq were filtered, followed by assembly of clean reads into transcript isoforms, using the *de novo* transcriptome assembler Trinity [27]. Subsequently, a BLASTX search was performed against the databases of non-redundant protein sequences (NR) and SwissProt with the criterion of *E*-value ≤10^−6^. Each unigene was annotated for protein and GO function based on the BLASTX alignment results.

### cDNA Synthesis and Reverse Transcription Polymerase Chain Reaction (RT-PCR) Amplification

First-strand cDNA synthesis was performed with M-MLV reverse transcriptase (Promega Corp., Madison, WI) and RNaseOUT (Invitrogen, Carlsbad, CA) according to the manufacturers’ protocols, using a mixture of random hexamer and oligo (dT)_20_ primers.

The RT-PCR amplification was conducted in a total reaction volume of 15 µl containing 150 ng of first-strand cDNA, 1X Super-Therm Gold Buffer, 0.4 µM of each primer, 2.5 mM MgCl_2_, 0.2 mM dNTP, 1X PCR enhancer CES [28], and 0.04 unit/µl of Super-Therm Gold DNA Polymerase (Bertec Enterprise, Taipei, Taiwan). The gene-specific primers used in the RT-PCR reactions were designed based on sequence information obtained from *de novo* transcriptome sequencing (forward-, 5′-CTAAGGATGAAGTGGACAGAGG-3′, and reverse-primer, 5′-GACCTGTGAGTCGTAAAAGGGT-3′). RT-PCR amplification was performed as follows: an initial step at 95°C for 10 min and an additional step at 97°C for 1 min; 30 cycles of 95°C for 30 s, 60°C for 45 s, and 72°C for 1 min; and a final extension at 72°C for 7 min. The RT-PCR products were visualized after 2% agarose gel electrophoresis and ethidium bromide staining under UV light.

### Quantitative PCR (qPCR) Analysis

Reverse transcribed cDNA samples (1∶5 dilution) from various developmental stages of *M. vitrata*, including larvae (4^th^–5^th^ instar), pupae (1–6 days), and adults (1–3 days), were amplified using the Rotor-Gene™ SYBR® Green Kit (Qiagen, Valencia, CA, USA) according to the manufacturer’s guidelines with the same primer set used for the RT-PCR. The real-time PCR was carried out on a Rotor-Gene 6000 (Corbett Life Sciences, Sydney, Australia) as follows: 5 min at 95°C, 40 cycles of 5 s at 95°C, and 10 s at 60°C, followed by a melting step at 60°C, temperature increasing to 95°C by 1°C every 5 s. β-actin (5′-CATCACCATCGGAAACGAAAGG-3′ and 5′-ATACTGTGTTGGCGTACAGGTC-3′), which has been reported to be relatively stable in expression across different simulations and developmental stages in insects [29]–[31], was chosen as an internal control gene to normalize the Marvi-DH-PBAN expression. A serial dilution of the cDNA mixture was used to produce a standard curve and the cDNA of 1-day-old female pupae served as the calibrator for gene expression analysis. A total of three qPCR replicates were performed for each sample. PCR amplification efficiencies and quantification cycle (*Cq*) values were calculated by the real-time PCR Miner program [32], and the relative Marvi-DH-PBAN gene expression at different developmental stages was evaluated using the Pfaffl method [33].

### Molecular and Phylogenetic Analyses of Sequence Data

The obtained raw sequence data were edited and assembled into contigs with ContigExpress of the Vector NTI Advance® program (Invitrogen, Carlsbad, CA). Nucleic acid sequences were translated into amino acid sequences, and open reading frames (ORFs) were identified using DNAsmac (http://biofreesoftware.com/dnasmac). Each sequence segment was aligned using the ClustalX2 application [34]. To determine intron-exon boundaries and intron phases, the Marvi-DH-PBAN cDNA sequence was subjected to ClustalX2 against the corresponding genomic DNA sequence. The last two nucleotides of an exon in a cDNA sequence are usually AG (adenine-guanine), the “shadow sequence” [35], [36]. These nucleotides are often mistakenly aligned with the last two bases of the intronic acceptor site, which are also AG in spliceosomal introns as a general rule, but other misalignment problems are not uncommon. Therefore, the intron phases were verified manually by inserting the intron position right before the upstream intronic donor base pair, GT (guanine-thymine), or immediately after AG at the end of the preceding exon. The evolutionary tree for interspecific and intraspecific phylogenetic relationships between DH-PBAN genes on the protein level was produced with MEGA5 [37].

## Results

### Structure of Maruca Vitrata PBAN Gene

**Figure 1:**
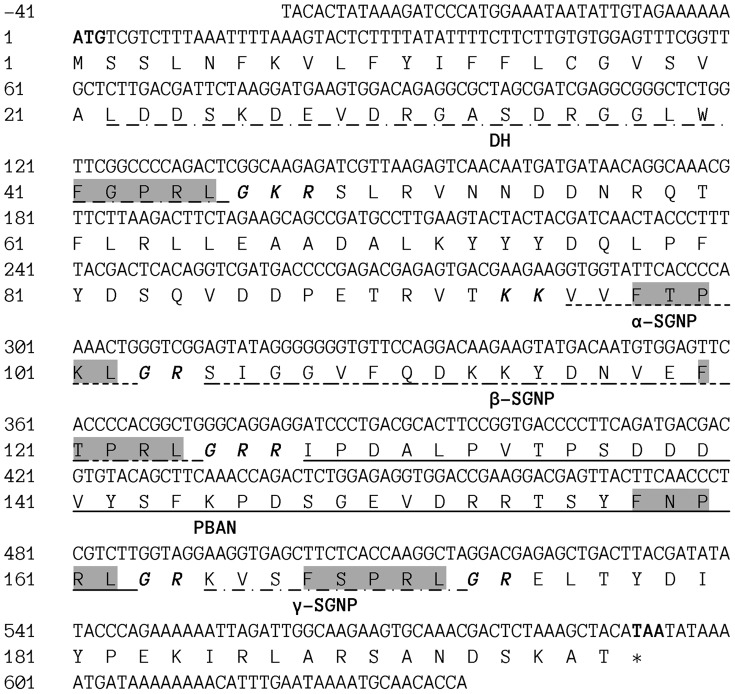
cDNA and derived amino acid sequence of Marvi-DH-PBAN. The nucleotide bases are numbered in the 5′ to the 3′ direction, and key elements of the gene are marked. The suggested start codon (ATG) and stop codon (TAA) are indicated in bold font. The five conjectural neuropeptides are underlined, and the dark shaded boxes represent the conserved FXPR/KLamide of each peptide. Predicted endoproteolytic cleavage sites are shown in bold italic letters.

Annotation of putative PBAN proteins was carried out using 92,001 assembled *Maruca vitrata* transcripts, retrieved from *de novo* transcriptome sequencing, as queries subjected to BLASTX against the National Center for Biotechnology Information (NCBI) non-redundant protein sequences (NR) and Swiss-Prot databases. A total of three transcripts returned BLAST results with sequence similarity to DH-PBAN in both databases (*E*-value ≤2.0×10^−23^; identity ≥57%). The further assembly of the three PBAN candidate homologs resulted in a unigene (GenBank ID: JQ901962; Figure 1). The full-length cDNA identified herein is composed of 677 bp, including a 5′-untranslated region (5′-UTR) of 41 bp, a 3′-untranslated region (3′-UTR) of 42 bp and an open reading frame (ORF). The ORF contains 591 nucleotides, encoding 197 amino acids.

Among the deduced amino acids, the *N*-terminal hydrophobic peptide from M^1^ to A^21^ was predicted as a potential signal peptide by SignalP 4.0 [38]. The Marvi-DH-PBAN transcript encodes five putative neuropeptides (*i.e*., DH, α-SGNP, β-SGNP, PBAN, and γ-SGNP) from 5′- to 3′-end, released at six possible endoproteolytic cleavage sites (G^46^-K^47^-R^48^, K^94^-K^95^, G^103^-R^104^, G^125^-R^126^-R^127^, G^163^-R^164^, and G^173^-R^174^), respectively [39]–[41]. The five precursor polypeptides share a common FXPR/KL fragment at their *C*-termini as shown in Figure 1.

**Figure 2:**
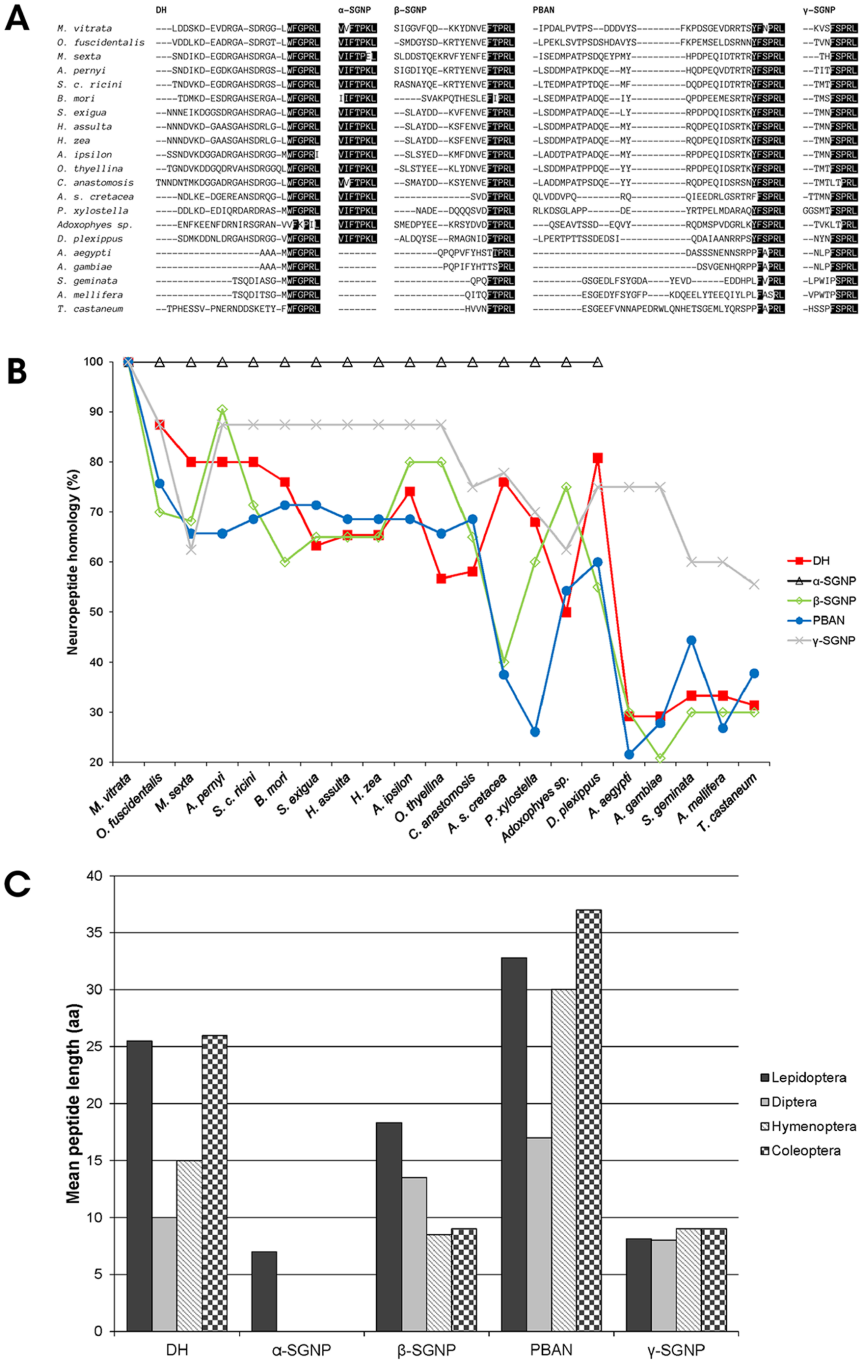
Comparative analysis of the homology of the five DH-PBAN neuropeptides. (A) Alignment of the FXPRLamide neuropeptide sequences of insect DH-PBAN genes. The five putative neuropeptides from the Marvi-DH-PBAN were compared with those of 15 other lepidopterans [*Omphisa fuscidentalis* (AFP87384), *Manduca sexta* (AAO18192), *Antheraea pernyi* (AAR17699), *Samia cynthia ricini* (AAP41132), *Bombyx mori* (AAB24327), *Spodoptera exigua* (AAT64424), *Helicoverpa assulta* (AAC64293), *Helicoverpa zea* (AAA20661), *Agrotis ipsilon* (CAA08774), *Orgyia thyellina* (BAE94185), *Clostera anastomosis* (ABR04093), *Ascotis selenaria cretacea* (BAF64458), *Plutella xylostella* (AAX99220), *Adoxophyes* sp. (AAK72980), *Danaus plexippus* (EHJ67284)], two dipterans [*Aedes aegypti* (Q16N80), *Anopheles gambiae* (Q7PTL2)], two hymenopterans [*Solenopsis geminata* (ADI88478), *Apis mellifera* (A8CL69)] and a coleopteran [*Tribolium castaneum* (EFA11568)]. Highly conserved *C*-terminal residues are shaded in black. **(B) A line chart comparing the amino acid sequence similarities of the five FXPRL peptides.** Percentages on the Y-axis are the homologies to *Maruca vitrata*. **(C) Comparison of average lengths of DH-PBAN peptides of species of four orders in Insecta.**

Figures 2A and 2B indicate that the Marvi-DH protein shows highest similarity to its ortholog of the bamboo borer, *Omphisa fuscidentalis* (Crambidae) (87.5%), and has the lowest similarity to DH proteins of mosquitoes *Aedes aegypti* and *Anopheles gambiae* (29.2%). The amino acid sequences of α-SGNPs are 100% identical among all compared lepidopterans, while α-SGNP is absent in Diptera, Hymenoptera, and Coleoptera. Marvi-β-SGNP is more divergent (20.8–90.5%), and Marvi-γ-SGNP more conserved among the different insect species (55.6–87.5%). The protein sequence of Marvi-PBAN is 75.7% similar to the PBAN protein of *O. fuscidentalis*, but only 21.6% homologous to that of the yellow fever mosquito, *A. aegypti*. Although translational and functional analyses have not yet been completed, we identified the isolated cDNA fragment as the DH-PBAN cDNA with high confidence based on sequence similarities. The lengths of the five neuropeptides were compared among the four insect orders, Lepidoptera, Diptera, Hymenoptera, and Coleoptera. Species of Lepidoptera and Coleoptera apparently have longer DH and PBAN than those of Diptera and Hymenoptera, while the γ-SGNP lengths of species in different orders were nearly identical, with lepidopterans having the longest β-SGNP (Figure 2C).

**Figure 3:**
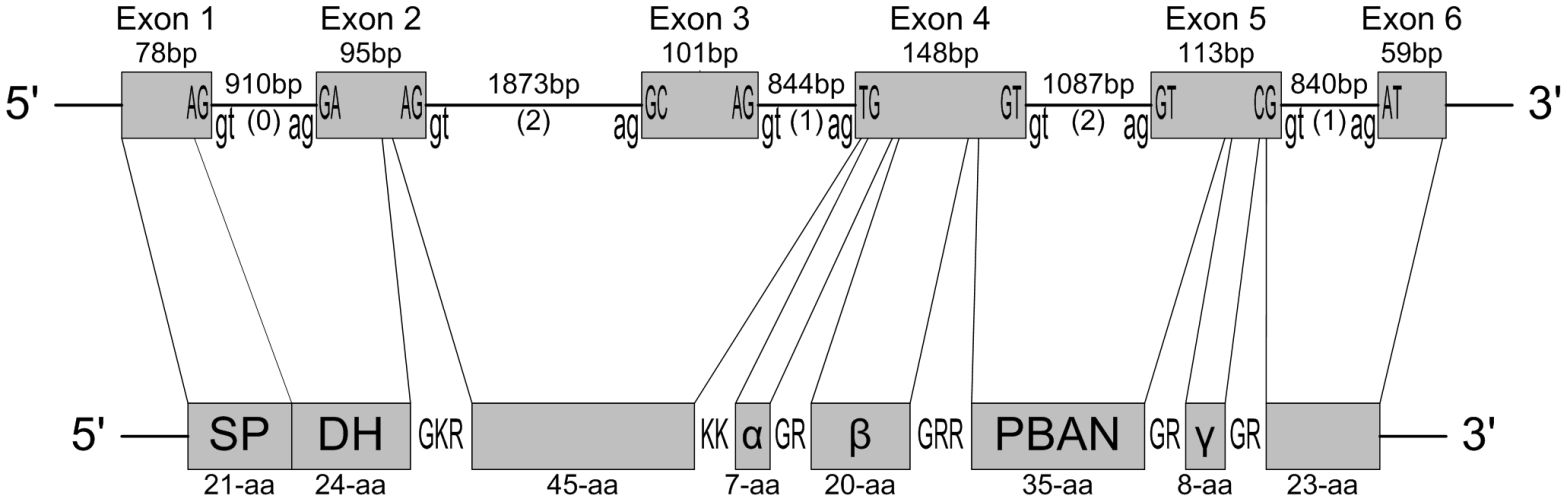
Schematic comparison of genomic DNA and the translated protein structures of Marvi-DH-PBAN. (represented by the top and bottom diagrams, respectively) Exons and peptides are shown as shaded boxes, and horizontal solid lines represent intronic regions with corresponding intron phase in parentheses. The actual size of each element was supplemented accordingly. Intron-exon boundary sequences are shown in the top figure with exon sequences in uppercase and intron sequences in lowercase. Letters between two peptides in the bottom Marvi-DH-PBAN polyprotein diagram indicate probable endoproteolytic cleavage sites. SP: signal peptide; DH: diapause hormone; α: α-SGNP; β: β-SGNP; PBAN: pheromone biosynthesis activating neuropeptide; γ: γ-SGNP.

Five primer pairs located within exons, each amplifying across a putative intron, were synthesized to retrieve the genomic sequence of Marvi-DH-PBAN based on the full-length cDNA sequence (Supporting Table S1). Potential intron-exon boundaries were predicted by the sequence alignments of the Marvi-DH-PBAN cDNA with homologs of the known DH-PBAN DNA sequences from *Clostera anastomosis* (EF614262), *Helicoverpa armigera* (AF492474), and *B*. *mori* (D16230). The 6,231 bp genomic DNA sequence of Marvi-DH-PBAN (GenBank accession number JX412479) is composed of six exons and five introns (Figure 3). The largest intron is 1,873 bp; the others ranged from 840–1,087 bp. The distribution of intron phases are phase 0, 2, 1, 2, and 1, respectively. All five introns are canonical introns, meaning that they exclusively possess 5′-GT-AG-3′ boundaries. The first two exons encode the Marvi-DH peptide, whereas Marvi-α-SGNP, β-SGNP, and partial PBAN are translated by Exon 4. The remaining Marvi-PBAN and γ-SGNP are encoded in Exon 5.

### Phylogenetic Relationship of DH-PBAN

**Figure 4:**
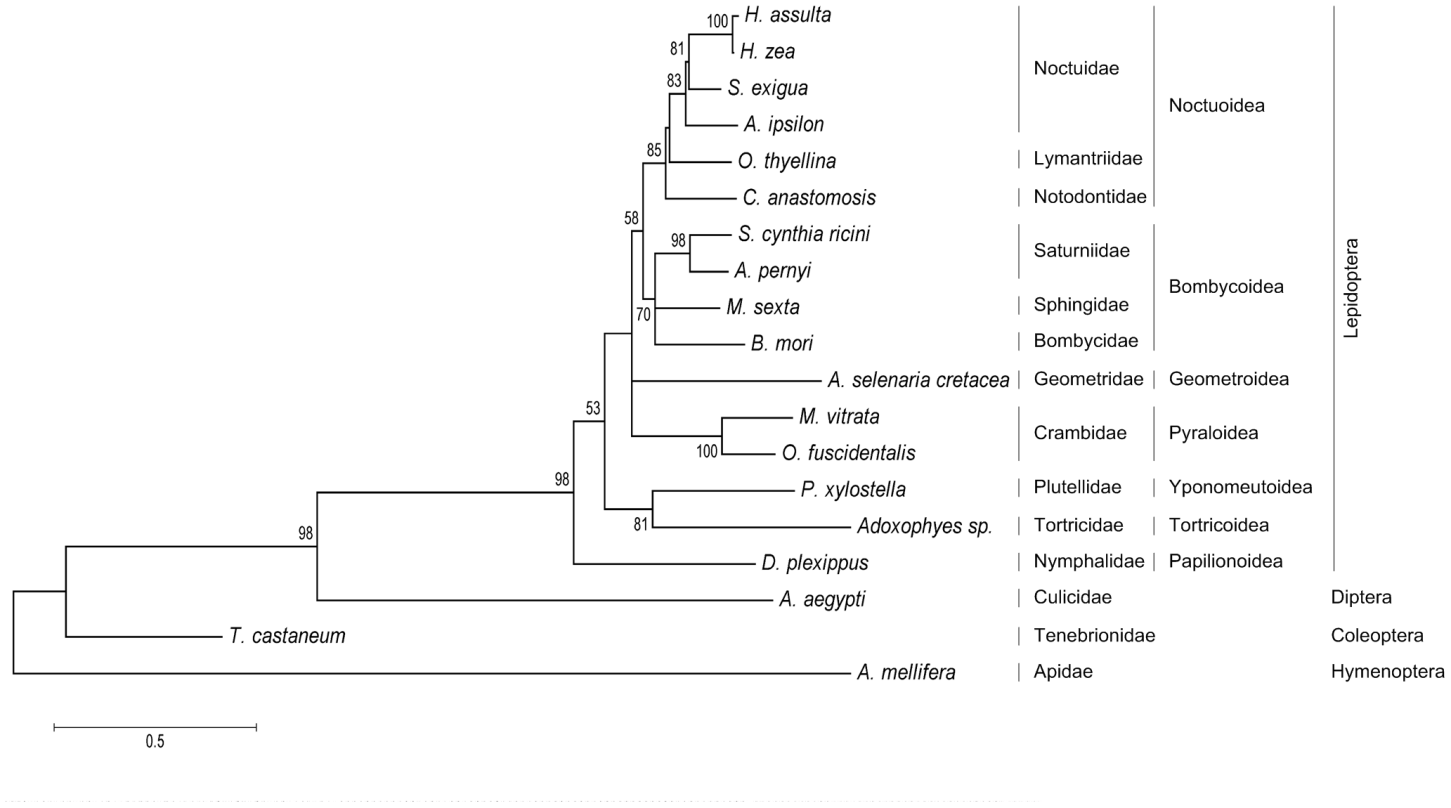
Evolutionary relationships of 19 known insect DH-PBAN amino acid sequences inferred using the neighbor-joining method. The phylogenetic tree was constructed based on a JTT matrix with 1000 bootstrap replicates by MEGA5 software. The percentages of bootstrap support above 50% are shown next to the branches. The tree is drawn to scale, with branch lengths in the units of the number of amino acid substitutions per site. The corresponding taxonomic families, superfamilies and orders of the taxa and clades are listed at the right.

The interspecific phylogenetic relationships based on the DH-PBAN ORFs from *M*. *vitrata* and the 18 other available insect DH-PBAN peptide sequences derived from GenBank are shown in Figure 4. The phylogram is consistent with the taxonomic classification of insects. *M. vitrata* was clustered together with *O*. *fuscidentalis* (Lepidoptera: Crambidae) with 100% bootstrap support. The members of the order Lepidoptera form a separate clade from the Diptera, Coleoptera and Hymenoptera of compared insects.

### Expression of Maruca Vitrata DH-PBAN Gene

**Figure 5:**
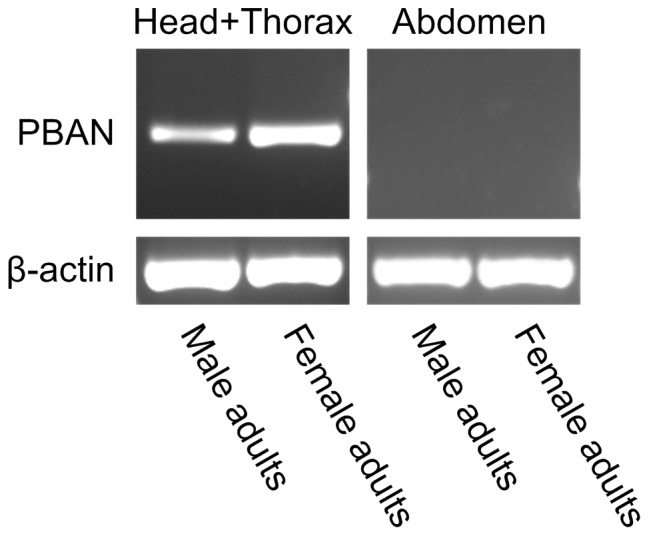
RT-PCR analysis for tissue distribution of Marvi-DH-PBAN mRNA. Total RNA extracted from the head plus thoracic segments (head+thorax) and abdominal tissues were reverse-transcribed to first-strand cDNA followed by PCR amplification. The bottom panel shows the amount of β-actin loaded per lane served as a control to verify equal loading for each sample.

The spatial distribution of Marvi-DH-PBAN transcripts was examined in head and thoracic complex and abdomen tissues from three-day-old adult males and females, which were previously reported to have the highest mating frequency [42] (Figure 5). RT-PCR amplicons of anticipated size of Marvi-DH-PBAN cDNA were observed in the head and thoraces from *M*. *vitrata* adults. However, no transcriptional signal was found in abdominal tissues of either sex, or else the expression level–thus amplified copy number–was too low to be detected by agarose gel electrophoresis.

**Figure 6:**
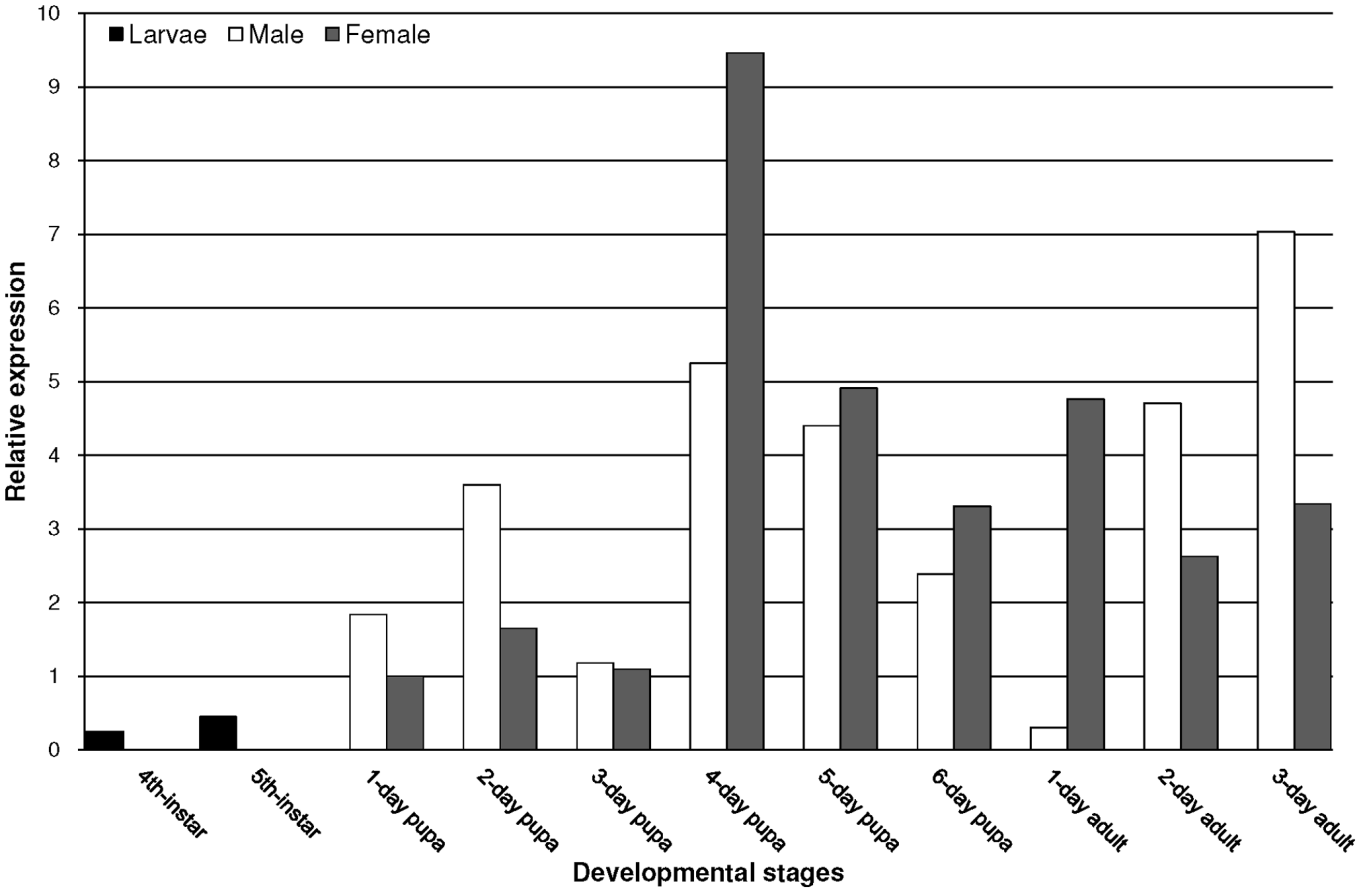
Developmental expression of the Marvi-DH-PBAN gene. The results are shown as the expression level relative to the female pupal (1-d pupae) DH-PBAN gene expression. The experiment was conducted for three times.

A real-time PCR analysis was performed to measure Marvi-DH-PBAN expression in different developmental stages of *M*. *vitrata*. Figure 6 shows that the relative number of transcripts was low in the 4^th^ and 5^th^ instar larvae. Over the pupal developmental stage, the DH-PBAN mRNA copy number was relatively low during the first three days, with higher expressions in males than in females, and then increased dramatically and reached its peak on day 4. From day 4 through day 6, expression of the gene gradually decreased while female pupae dominated male pupae in the expression level. The gene expression of Marvi-DH-PBAN decreased immediately after the adult emergence in males, but was elevated promptly from day 2 until day 3. On the contrary, DH-PBAN expression in female moths remained at a consistent level with that of the late pupal stage and subsequently decreased on day 2 and day 3.

## Discussion

Sex pheromone traps play an important role in pest management as an environmentally safe bio-control means, and the genes involved in the biosynthesis of sex pheromone in insect pests have been of great interest to entomologists. Although the structural and functional properties of DH, PBAN and the other PBAN/pyrokinin family members have been studied extensively in insects, the data in the present study are the first in which the genomic organization and phylogenetic characterization of DH-PBAN have been documented for an insect species in the family Crambidae. We have successfully isolated a full-length DH-PBAN cDNA from *M*. *vitrata*. The 677 bp transcript includes an ORF that encodes the DH-PBAN protein of 197 amino acids. The structural organization of the DH-PBAN polyprotein is highly conserved among lepidopterans. Moreover, the phylogenetic analysis of DH-PBAN genes also matches the phylogeny and evolutionary diversity in insects.

The five FXPRL neuropeptides released from the Marvi-DH-PBAN precursor were cleaved at six potential endoproteolytic cleavage sites that, except for the K-K site, share a conserved sequence of G-(K/R)-R (Figure 1). Whereas the basic arginine residue is served as a canonical signal preceding the cleavage sites [43], the presence of the glycine residue further indicated amidation of the *C*-terminus of neuropeptides in Marvi-DH-PBAN, which is pivotal for the biological activity of peptide hormones [44]. The cleavage motif of basic K-K residues has been reported to occur less frequently in neuropeptide precursors; however, it was observed being cleaved in 64% of its occurrence in *Apis mellifera* and *Drosophila melanogaster* [41]; thus it was not surprising to find it present in the Marvi-DH-PBAN.

We have confirmed the molecular structure similarity of known DH-PBAN genes among species in the order Lepidoptera (Figure 2A, B). Within Lepidoptera, the full protein sequence of Marvi-DH-PBAN is most homologous to DH-PBAN from *O*. *fuscidentalis* of the family Crambidae (83%), and least similar to that of *Ascotis selenaria cretacea* in the Geometridae (63.9%). Figure 2B shows that neuropeptide homology decreased dramatically when comparing Marvi-DH, β-SGNP, PBAN, and γ-SGNP with those from members of the Diptera, Hymenoptera, and Coleoptera. The DH neuropeptide was observed to have the most conserved *C*-terminal motif, WFGPRL, of the DH-PBAN polyprotein throughout insect species (Figure 2A), and it has been reported to be functional in two opposing effects: inducing embryonic diapause in *B*. *mori* [6], [45], [46], and ceasing pupal diapause in moths from Noctuidae [47]–[51]. The function of Marvi-DH remains unrevealed; however, it is noteworthy that DH peptides from Crambidae (Pyraloidea) members lack a histidine between residues A^33^-S^34^, which is exclusively conserved in closely related species from Bombycoidea and Noctuoidea, something that has not been previously reported. A further comparison among organisms of the three super-families may advance our understanding of the structural and functional variations of DH neuropeptide.

α-SGNP, missing in Diptera, Hymenoptera, and Coleoptera, has a peptide sequence similarity to leukopyrokinin, which stimulates the hindgut muscle contraction in *Leucophaea maderae* [52]. Although it is not clear whether Marvi-α-SGNP performs a similar function, as α-SGNP is identically conserved across lepidopteran species (suggesting essential function within this peptide), it is plausible that the vital functional domain had been excised from the original locus, and was subsequently incorporated into the DH-PBAN gene sequence through gene shuffling [53], [54]. Alternatively, we may hypothesize that the novel primordial α-SGNP gene fragment was horizontally transferred into and has been fixed in ancestral strains of the relatively young order Lepidoptera (arising in the Eocene epoch; see review by [55]), and passed to all descended lineages, under positive selection over the insect evolutionary history (see review in analogous issue by [56]). Choi and Vander Meer [16] proposed another theory in which the α-SGNP domain was fused with β-SGNP resulting in a single neuropeptide in fire ants. These hypotheses require more evidence for support from future studies.

β-SGNP was shown to be the most divergent among the five peptides deduced from DH-PBAN genes (Figure 2B). It is not uncommon for peptide sequences to have evolved either gain or loss functional domains to adapt to environmental changes. This is known, for example, even among closely related taxa [57], [58]. The sequence of Marvi-γ-SGNP provided the highest similarity to those from the Pyraloidea, Bombycoidea, and Noctuoidea (87.5%), excluding *Manduca sexta* (62.5%), which is one amino acid shorter, and *C*. *anastomosis* (75%), which possesses a variant *C*-terminal pentapeptide sequence, LTPRLamide.

In the PBAN protein, the YFSPRL motif is highly conserved within lepidopteran species (Figure 2A). Intriguingly, the third residue of this domain (serine) was substituted in *M. vitrata* with asparagine, each of which possesses polar neutral side-chains but with distinct hydrophobicity. Up to now, this substitution has been found only in *M. vitrata* in the present study. Lassance *et al.* [59] reported different sex pheromone blend ratios as a result of the presence of multiple allele forms at a fatty acid reductase gene in the European corn borer, *Ostrinia nubilalis*, which led to the formation of reproductively isolated races. Because the PBAN protein is commonly known to associate with the stimulation of pheromone synthesis, we posited that such a substitution in the Marvi-PBAN gene may have some impact on pheromone production, and play a role in the evolution of races within *M. vitrata*. However, further validation of the correlation between the residue substitution in PBAN and *M. vitrata* race evolution awaits phylogenetic analysis of large populations.

It is interesting to note that in Figure 2C the lengths of the PBAN/pyrokinin family of peptides in Lepidoptera are generally longer than those of the compared orders. Since Lepidoptera is the most diverse order of insects [60] and DH-PBAN is closely associated with sexual behavior and reproduction in moths, neuropeptide length variation among insect orders may partially reflect insect diversity. Because peptide length is expected to be positively proportional to the number of functional motifs or sites and to the mutation rate [61], [62], which is a key source of evolution and speciation, it is plausible that lengthy DH-PBAN neuropeptides in lepidopterans resulted in their abundant speciation events. This inference is further supported by the species richness of the compared orders. Whereas Diptera, with the shortest DH-PBAN peptides, has only 125,000 species, there are approximate 350,000 species in Coleoptera, 160,000 species in Lepidoptera, and 150,000 species in Hymenoptera [63], [64]. In particular, there is a statistically significant relationship of mean peptide length of both DH and PBAN when compared to the biodiversity of the four orders (*i.e.*, Coleoptera>Lepidoptera>Hymenoptera>Diptera; χ^2^ = 9.928, *df* = 3, *P*<0.05 and χ^2^ = 8.969, *df* = 3, *P*<0.05, respectively, Kruskal-Wallis test). It could be postulated that the PBAN/pyrokinin neuropeptide size may therefore be correlated with species diversity in Insecta but further investigation is necessary to confirm this relationship.

The genomic locus of Marvi-DH-PBAN contains five introns, all spliced following the “GT-AG” rule (Figure 3), consistent with the genomic structure reported in *B. mori* [65], *H. armigera* [66], and *C. anastomosis* [67]. In addition, all the Marvi-DH-PBAN introns share the same intron positions and phases as those observed in *B. mori*, *H. armigera*, and *C. anastomosis*. These results support previous studies that indicate introns maintain their position in most lineages over long evolutionary periods (see review by [68]). Previous studies have indicated that coding regions conserved the general junction sequences of 5′-A/CG|G-3′ (where the annotation “|” refers to the intron position) of the intron-exon boundaries in eukaryotes [69], [70]. Of all six exons in the Marvi-DH-PBAN gene, Exon 1, 2, 3 and 5 match the pattern perfectly, whereas Exon 4 and 6 exhibit a variety of the exon sequences that may be ascribed to the evolutionary process.

The phylogenetic construction, consisting of the majority of known DH-PBAN protein sequences across four orders (Lepidoptera, Diptera, Coleoptera and Hymenoptera), was carried out in the present study using the neighbor-joining method (Figure 4). Examined species from the same genus, *Helicoverpa assulta* and *H. zea* are monophyletic. Without exception, members from the same family formed monophyletic clades (Noctuidae, Saturniidae, and Crambidae). The families Noctuidae, Lymantriidae and Notodontidae form a monophyletic clade comprising the superfamily Noctuoidea. The sister group to the Noctuoidea is the Bombycoidea, to which the families Saturniidae, Sphingidae and Bombycidae belong. The Geometroidea (*Ascotis selenaria cretacea*) and the Pyraloidea form a trichotomy with the Noctuoidea plus Bombycoidea clade. The sister taxon to the above clade is the Yponomeutoidea plus Tortricoidea and the basal clade of the Lepidoptera is the Papilionoidea. Successive out-groups to the Lepidoptera are the Diptera (*Aedes aegypti*), the Coleoptera (*T. castaneum*), and the Hymenoptera (*Apis mellifera*). On the whole, the DH-PBAN protein sequence similarity is correlated with the basic taxonomic relationships among the species and infers the feasibility and sensitivity of the DH-PBAN gene sequences as a phylogenetic marker in class Insecta. The placement of the Papilionoidea (*Danaus plexippus*) at the most basal group of the Lepidoptera, and thus a sister group to the other super-families of the order, is unexpected [71] and poses some questions, raising the likelihood that the Lepidoptera might be polyphyletic.

Thus far, the DH-PBAN neuropeptides have been known to be primarily synthesized in and released from the subesophageal ganglion (SG) in moth species. In the present study, the DH-PBAN transcripts were exclusively located in the head and thorax of adult male and female *M. vitrata*, but no expression was detected in the abdomen of adults (Figure 5). Our results do not conflict with published results that the DH-PBAN gene is also feebly expressed in thoracic ganglia of *H. zea* [72], *H. virescens* [50], *Samia cynthia ricini* [73], *M. sexta* [74], *Plutella xylostella* [75], *Ascotis selenaria cretacea* [76], *Antheraea pernyi* [77], and *Solenopsis invicta* [78]. Association between DH-PBAN expression and abdominal tissue distribution has been reported only in the FXPRLamide-like immunoreactivity assays in abdominal ganglions [73], [77]–[79]. Hence, further investigation using immunostaining techniques seems necessary not only to confirm the DH-PBAN peptide location but also to shed light on the PBAN transport path from the SG to the pheromone gland in female moths.

The temporal gene expression profile of DH-PBAN over different developmental stages of insects in the family Crambidae has not been analyzed previously. Here we report, for the first time, the gene expression changes of DH-PBAN during *M. vitrata* development (Figure 6). Based on the results of qPCR analysis, the Marvi-DH-PBAN mRNA is expressed at a relatively low level during late larval stages. Much higher expressions of the Marvi-DH-PBAN gene were observed during the mid- and late-pupal phase, similar to the mode observed for *S. cynthia ricini* [73] and non-diapausing *A. pernyi* [77], but contrary to those that have been reported in *C. anastomosis* [67], diapausing *M. sexta* [74] and diapausing *A. pernyi* [77]. As it is commonly known that growth and differentiation occur within the chrysalis, the gene expression pattern at this stage suggests that the Marvi-DH-PBAN protein may be involved in the stimulation and regulation of adult development in pupae, which is consistent with the physiological function of DH-PBAN in stimulating adult development and formation in *H. virescens* and *H. armigera* pupae [48]–[51].

In adult *M. vitrata* females, the Marvi-DH-PBAN mRNA expression is greater on the first day than on day 2 and 3, which coincides with the timing of the activation of sex pheromone synthesis for mating attraction. It has already been affirmed that the pheromone production is significantly higher in virgin moths than mated moths [80], [81]. When mated *Heliothis subflexa* and *H. virescens* females injected with PBAN, they produced pheromone at levels and ratios comparable to those produced by virgin females [82], which confirmed the higher expression of PBAN in newly emerged female moths. Interestingly, the Marvi-DH-PBAN expression was moderately decreased in day 2 and 3 in adult samples from female cDNA, whereas the level of DH-PBAN mRNA in male moths increased dramatically from day 2 to day 3, an event that has never before been reported in the literature. As the pheromonotropic function of PBAN might not be expected in male moths, and that *M. vitrata* does not undergo diapause [83], FXPRL peptides may also participate in many other bioactivities in addition to the stimulation of pheromone biosynthesis (see reviews by [8], [10]). Our finding here may in turn imply the extra need for male *M. vitrata* to exploit various physiological effects, modulated by FXPRLamides, to pinpoint their mating targets and/or for other mating behaviors in the field shortly after the release of sex pheromone from females. For instance, in *H. armigera*, PBAN was demonstrated to up-regulate fatty-acid and alcohol components, correlating with chemical communication systems and behavioral responses, in hair-pencil-aedaegus complexes. This is a male-specific tissue structurally homologous to the female pheromone gland, and the timing of regulation was coincident with female pheromone production [84]. Due to the fact that the full DH-PBAN gene undergoes transcription even if only one or a few of the five neuropeptides are present, a striking and dominant expression level of DH-PBAN transcript in male moths of *M. vitrata* is evident. Moreover, the dominant expression level of DH-PBAN in *M. vitrata* females from day 4 of the pupal stage may to some extent be correlated with the regulatory mechanism of PBAN in order for rapid stimulation and synthesis of the sex pheromone soon after adult eclosion. Additional study of Marvi-DH-PBAN at pre- and post-translational levels is required to validate this hypothesis and to understand its biological functions.

## Conclusions

The present study provides several new clues about DH-PBAN. The genomic DNA sequence of DH-PBAN was for the first time elucidated in a Crambidae species, *M. vitrata*, along with its transcript cDNA sequence encoding putative protein. A single residue substitution of the *C*-terminal FXPRLamide in the PBAN peptide was found only in *M. vitrata*, which could be a valuable phylogenetic marker for differentiating *M. vitrata* from other insects. As insect metamorphosis and pheromonotropic activity are believed to be regulated by DH-PBAN, it is anticipated that developmental and reproductive behavior in *M. vitrata* could be disrupted by interference with DH-PBAN at the DNA or protein level, which in turn hindered the stimulating physiological responses. For example, the RNA interference (RNAi) pathway was shown to be capable of inducing larval lethality in coleopterans through intake of dsRNA expressed in the host plants [85], and this technique may be introduced to silence the DH-PBAN gene expression in *M. vitrata* and achieve more environmentally friendly methods of pest management. Furthermore, the existence of population variation in sex pheromone component responses observed in *M. vitrata* has increased the difficulty of pest control. We are hopeful that the fundamentals of Marvi-PBAN indicated in this study can be employed in future studies to clarify the relation of PBAN to specific pheromone blends in various geographical regions and relevant biosynthesis pathways.

## Supporting Information

Table S1 **List of primer sequences.** Primer pairs used in the current study for amplification of intronic sequences in the Marvi-DH-PBAN gene.
(DOCX)
