# Supplementary material for: Identification and Expression Analysis of Diapause Hormone and Pheromone Biosynthesis Activating Neuropeptide (DH-PBAN) in the Legume Pod Borer, Maruca vitrata Fabricius
Source: PLoS One. 2014 Jan 7;9(1):e84916. doi: 10.1371/journal.pone.0084916 (PMC3883689; doi:10.1371/journal.pone.0084916)
Supplement: Table S1 — List of primer sequences. Primer pairs used in the current study for amplification of intronic sequences in the Marvi-DH-PBAN gene. (DOCX) [file pone.0084916.s001.docx]

**Table S1. List of primer sequences.** Primer pairs used in the current study for amplification of intronic sequences in the Marvi-DH-PBAN gene.

| **Oligo title** | **Sequences (5’ to 3’)** |
| --- | --- |
| PBAN_Intron1_F | TTCTTCTTGTGTGGAGTTTC |
| PBAN_Intron1_R | GTTGACTCTTAACGATCTCTTG |
| PBAN_Intron2_F | GACTCGGCAAGAGATCGTTAAGAG |
| PBAN_Intron2_R | GACCTGTGAGTCGTAAAAGGGTAG |
| PBAN_Intron3_F | CTACCCTTTTACGACTCACAGGTC |
| PBAN_Intron3_R | CATACTTCTTGTCCTGGAACACCC |
| PBAN_Intron4_F | GGGTGTTCCAGGACAAGAAGTATG |
| PBAN_Intron4_R | CAAGACGAGGGTTGAAGTAACTCG |
| PBAN_Intron5_F | GAAGGACGAGTTACTTCAAC |
| PBAN_Intron5_R | TGTAGCTTTAGAGTCGTTTG |
